# Prognostic markers in patients with chronic lymphocytic leukaemia on targeted therapy, chemoimmunotherapy with anti-CD20 monoclonal antibody: a systematic review and meta-analysis of prognostic factors

**DOI:** 10.1186/s12885-022-10223-0

**Published:** 2022-11-25

**Authors:** Zekhethelo A. Mkhwanazi, Tawanda M. Nyambuya, Snenhlanhla A. Mfusi, Bongani B. Nkambule

**Affiliations:** 1 School of Laboratory Medicine and Medical Sciences (SLMMS), College of Health Sciences, University of KwaZulu-Natal, Durban, South Africa; 2 Department of Health Sciences, Faculty of Health and Applied Sciences, Namibia University of Science and Technology, Windhoek, Namibia

**Keywords:** Chronic lymphocytic leukaemia, Prognosis, Chemoimmunotherapy, Anti-CD20, Targeted therapy

## Abstract

Combination chemoimmunotherapy (CIT) consisting of anti-CD20 has improved the progression-free survival (PFS) and overall survival (OS) of patients with chronic lymphocytic leukaemia (CLL). We performed a comprehensive synthesis of prognostic factors in patients with CLL on combined CIT with anti-CD20 antibodies compared with standard chemotherapy alone or targeted therapy.

We searched the MEDLINE and academic search complete electronic databases as well as clinicaltrials.gov (from inception up to 01 August 2022) for randomised controlled trials examining chemoimmunotherapy and targeted therapy in patients with CLL. The risk of bias and the quality of evidence was assessed using the quality in prognostic studies tool (QUIPS).

A total of 10 prognostic factors were identified and evaluated in patients with CLL on anti-CD20 antibody-containing CIT. The predictive value of the following prognostic factors was confirmed and associated with poor patient outcomes; deletion 17p (HR = 3.39), Immunoglobulin heavy chain variable region gene mutation status (HR = 0.96) and β_2-_microglobulin (HR = 1.41).

Conventional predictive factors may have retained prognostic value and could be useful in the stratification of patients who may be non-responsive to CIT.

**Trial registration: **International Prospective Register of Systematic Reviews (PROSPERO) registry (CRD42021218997).

**Supplementary Information:**

The online version contains supplementary material available at 10.1186/s12885-022-10223-0.

## Introduction

The prevalence of chronic lymphocytic leukaemia (CLL) in adults over the age of 65 has gradually increased in high income countries [1, 2]. CLL disproportionately affects males, and an inferior survival rate in males has been reported in several studies [3–5].

Over the last two decades, novel clinical and genetic-based prognostic factors have been identified in patients with CLL [6]. These include age, gender, immunoglobulin heavy chain variable region gene (*IGHV*) mutation status and cytogenetic abnormalities [7, 8], the aberrant expression of CD38 and ZAP70 [9], *TP53* mutation [10], β_2_-microglobulin [11], and the Eastern Cooperative Oncology Group (ECOG) performance status [6, 7]. The development and implementation of prediction models have allowed for the risk-stratification of patients with CLL based on genetic traits [12].

In patients with CLL, therapy consisting of ibrutinib [13, 14], chlorambucil [15], fludarabine and cyclophosphamide [16, 17] yielded low overall response rates (ORR), with treated patients having an estimated 5-year overall survival (OS) of < 40% [18, 19]. These clinical outcomes in patients with CLL led to a shift towards novel antibody-based therapies in the last decade. These include rituximab, an anti-CD20 monoclonal antibody which when administered in combination with standard chemotherapy, improves the patient response rates and is associated with complete remission (CR) in patients with CLL [20–22]. However, despite the benefit of chemoimmunotherapy (CIT) with rituximab, patient outcomes are highly variable [23]. The efficacy of rituximab-based CIT has been demonstrated in cohorts of patients without the associated genetic aberrations such as Del(17p) and TP53 mutations [24].

The advances and refinement of prognostic risk scores has led to improved risk stratification of patients with CLL. The cornerstone of these risk scores, are the revised Rai [25] and Binet [26] staging systems, and novel prognostic indices such as CLL International Prognostic Index (CLL-IPI) [27] which allow for a precise risk stratification. Pertinent challenges in the risk stratification of patients with CLL on CIT include the lack of cumulative evidence on the predictive value of integrated cell and genetic based prognostic models [28]. Moreover, the lack of diverse multi-ethnic cohorts and prevalent risk factors also contribute to the imprecision of these predictive models [29, 30]. Therefore, the current systematic review and meta-analysis sought to identify and evaluate studies reporting on prognostic factors in patients with CLL on CIT or targeted therapy. Moreover, we aimed at providing a comprehensive synthesis and confirmation of prognostic factors associated with poor clinical outcomes in patients with CLL on CIT.

## Methods

### Eligibility criteria

The eligibility criteria was based on the Population, Index prognostic factor, Comparator prognostic factors, Outcome, Timing and Setting (PICOTS) guidelines [31]. We included randomised controlled trials (RCTs) reporting on prognostic factors in patients with CLL on CIT containing anti-CD20 monoclonal antibodies (rituximab, obinutuzumab, ofatumumab) or targeted therapy such as Bruton’s tyrosine kinase (BTK) inhibitors. We also included studies that aimed at developing or validating predictive models for mortality in CIT-treated patients with CLL. In addition, we included studies reporting on predictive measures at any time point and setting. Reviews, letters, and case-studies were excluded. In this systematic review, predictive models were considered as multivariable models used to predict survival in patients with CLL using selected predictors. We considered index prognostic factors derived from the CLL International Prognostic Index (CLL-IPI) [27], the German CLL Study Group (GCLLSG) [32], and the MD Anderson Cancer Centre (MDACC) nomogram predictive models [33].

### Search strategy and selection process

A systematic literature search was performed by two independent reviewers (ZAM and BBN) on the MEDLINE, MasterFILE premier, Health source: Nursing/Academic edition, and clinical trials.gov. We made use of Medical Subject Headings (MeSH) and related synonyms which included, chronic lymphocytic leukaemia, rituximab, ofatumumab, Obinutuzumab, anti-CD20 monoclonal antibody, ibrutinib, venetoclax, acalabrutinib, idelalisib and prognosis. All electronic databases were searched from inception to the 1^st^ of August 2022. A detailed search strategy is presented in Supplementary Table 1. To augment the database search, we screened the bibliographies of relevant reviews and included studies.

### Data extraction

Two reviewers (ZAM and BBN) independently extracted data items from the included studies defined in the critical Appraisal and data extraction for systematic Reviews of prediction Modelling Studies for Prognostic factors CHARMS-PF checklist [34]. The extracted study characteristics included, source of data, participant description, sample size, outcomes to be predicted, candidate predictors, type of model.

### Risk of bias and quality assessment

The certainty and strength of the evidence was assessed by two independent reviewers (ZAM, SAM) using the Quality In Prognostic Studies (QUIPS) tool [31]. The tool consists of six domains used to appraise studies of prognostic factors (Supplementary Table 2). A third reviewer (BBN) was consulted for arbitration.

### Statistical analysis

The Cohen’s kappa was used to assess the inter-rater reliability for the study selection and the study quality and risk of bias assessments [35]. The hazard ratios (HR) or odds ratios (OR) and 95% confidence interval (CI) were pooled to estimate the pooled OS and PFS. The effect estimates of studies were pooled using a random-effects model [36]. The *I*^*2*^ and Chi squared statistical tests were used to assess the levels of statistical heterogeneity [37, 38]. An *I*^*2*^ value of  >50% was considered as substantial [36]. All data analysis was performed using STATA 16.0 (StataCorp LP, TX, USA).

### Subgroup and sensitivity analyses

To explore the sources of heterogeneity amongst the included studies, we performed a sensitivity analysis based on the study design and quality.

### Confirmation of predictive factors

The reported prognostic factors were confirmed based on the robustness of the overall direction of the effect across all eligible studies. Moreover, adjusted effect estimates that remained statistically significant (*p* < 0.05) after adjusting for covariates in the multivariate analysis were considered as confirmed.

## Results

### Included studies

**Fig. 1:**
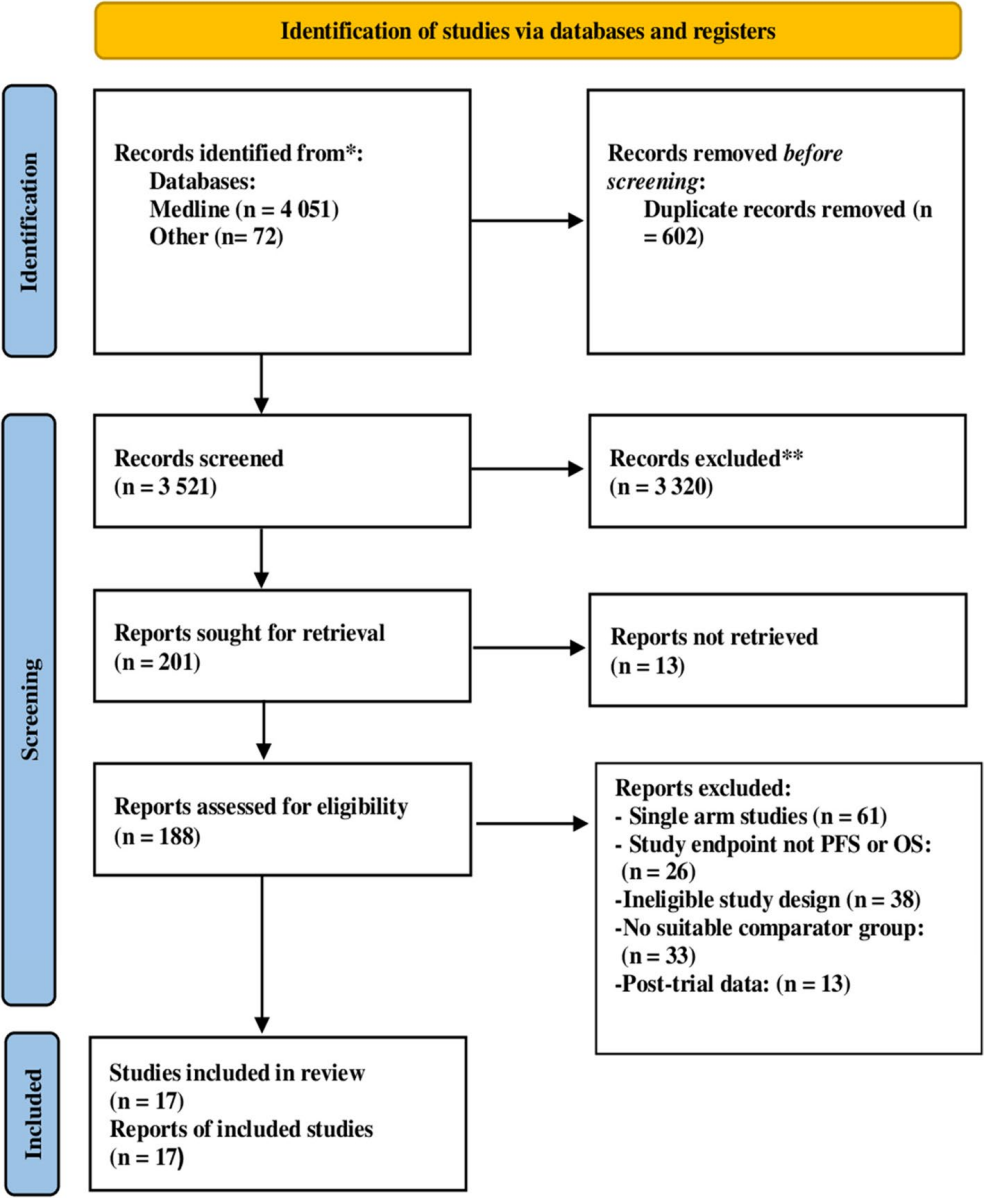
PRISMA flow diagram showing the study selection process

We retrieved a total of 4123 citations through the database search, and after excluding 602 duplicated studies only 3521 studies were eligible for screening. Amongst these, 3320 studies were ineligible and excluded during the abstract screening phase. A total of 201 citations were retrieved and 118 articles with available full-texts were assessed for eligibility. A total of 171 studies were excluded for the following reasons: single arm studies (*n* = 61), ineligible study design (*n* = 38), clinical endpoint not reported (*n* = 26); no suitable comparator group (*n* = 33); only contained post-trial follow-up data (*n* = 13). In all, 17 studies [14–17, 39–51] met the inclusion criteria and were included in the qualitative and quantitative analysis (Fig. 1). The overall reviewer agreement for study selection, was 89% (kappa = 0.82).

### Characteristics of included studies

The 17 included studies were published between 2010 and 2021 comprising of a total of 7 349 patients with CLL (Table 1). Most of the included trials were multicentre studies and the study sample size varied from 66 to 817 patients (Median: 389, IQR: 296—532). The age of enrolled participants ranged from 22 – 92 years.

**Table 1:** Characteristics and outcomes of included CLL studies (*n* = 17)

Study	Geographic region	Aim	Staging	Model performance	Outcome; Adjusted effect estimate;	Main Findings
Robak et al. 2010 [16]	Europe	To compare CIT with fludarabine, cyclophosphamide and rituximab (FCR) with standard chemotherapy (FC) in patients with previously treated CLL	Binet Stage A, B and C	No	PFS; HR: 0.65, OS; HR: 0.83	CIT with rituximab improved a 2-year PFS. Patients with poor prognostic factors such as del11q, unmutated *IGHV*, or positive ZAP-70 benefited from FCR
Hallek et al. 2010 [17]	Europe	To investigate whether adding rituximab to chemotherapy with FC would improve the survival outcomes of treatment-naïve, physically fit patients with CD20^+^ CLL	Binet Stage A, B and C	No	PFS; HR: 0.56, OS; HR: 0.67	The addition of rituximab to chemotherapy improved 3-year PFS and OS and resulted in significantly higher PFS in most genetic subgroups including del(17p), del(11q), del(13q) and trisomy 12. An improvement in PFS was noted in all disease stages
Goede et al. 2014 [15]	Europe	To determine whether CIT with rituximab or obinutuzumab would be beneficial in previously untreated patients with CLL and comorbidities	Binet stage C, symptomatic disease	No	PFS; HR: 0.44, OS; HR: 0.66 (R-Chl vs Chl) PFS; HR: 0.18, OS; HR: 0.41 (O-Chl vs Chl)	CIT with rituximab or obinutuzumab resulted in a better response and prolongation of a 2-year PFS as compared to treatment with chlorambucil alone
Chanan-khan et al. 2016 [46]	Europe, Americas, Asia	To assess the efficacy and safety of ibrutinib versus placebo in combination with bendamustine plus rituximab in patients with relapsed or refractory chronic lymphocytic leukaemia or small lymphocytic lymphoma	Binet stage A, B and C or Rai stage 0-II and III/IV	No	PFS; HR: 0.203, OS; HR: 0.577	Addition of ibrutinib to CIT resulted in significant improvement in PFS as compared to CIT alone in patients with R/R CLL and having high-risk features such as unmutated IGHV status, del(11q), and bulky disease
Hillmen et al. 2015 [47]		To investigate whether the addition of ofatumumab to chlorambucil could lead to better clinical outcomes than does treatment with chlorambucil alone	Binet stage A, B and C	No	PFS; HR: 0.91, OS; HR: 0.57	Addition of ofatumumab led to a significant benefit in progression-free survival in most subgroups of patients
Van Oers 2015 [51]		To compare ofatumumab maintenance treatment with observation for patients in remission after re- induction treatment for relapsed CLL	Binet stage A, B and C	No	PFS; HR: 0.50, OS; HR: 0.85	Ofatumumab maintenance improved PFS in patients with relapsed CLL
Greil et al. 2016 [44]	Europe	To investigate the potential of rituximab maintenance therapy to improve survival outcomes in patients with CLL who respond to rituximab-containing induction regimen	Rai stage 0/I/II or stage III/IV	Yes	PFS; HR: 0.50 OS; HR: 0.77	Rituximab maintenance therapy prolonged a 3-year PFS. The effect of rituximab on PFS was comparable across prognostic factors analysed. OS was not reached in both the rituximab and observation group due to shorter follow-up time
Robak 2017 [42]	18 countries	To investigate the potential of adding ofatumumab to FC to improve PFS in relapsed CLL	Rai stage 0-II or stage III/IV	No	PFS; HR: 0.67 OS; HR: 0.78	Addition of ofatumumab to chemotherapy with FC improved PFS compared to FC alone in patients with relapsed CLL
Dartigeas et al. 2017 [45]	Europe	To compare maintenance treatment with rituximab vs. no further treatment to prolong PFS in treatment-naive and fit patients aged ≥ 65 years with CLL	Binet stage B or C	Yes	PFS; HR: 0.55, OS; HR: 0.89	Maintenance therapy with rituximab improved 3-year PFS as compared to observation. OS was not reached in both groups at the time of analysis
Robak et al. 2018 [43]	Europe	To assess the effect of maintenance treatment with rituximab vs. no further treatment in previously untreated patients with progressive CLL	Rai stage I-IV	No	PFS; HR: 0.418	A 3-year PFS was significantly longer in the maintenance arm compared to the observation arm
Woyach et al. 2018 [14]	Americas	To evaluate the efficacy of ibrutinib, either alone or in combination with rituximab in older patients with untreated CLL	Intermediate to high-risk modified Rai stage disease	No	PFS/OS; HR: 1.06	There was no significant difference in 2-year PFS and OS between the two arms. Interactions between cytogenetics and effect of treatment on PFS were observed
Seymour et al. 2018 [40]	Americas, Europe	To evaluate the efficacy of venetoclax in combination with rituximab in patients with relapsed or refractory CLL	Not stated	No	PFS; HR: 0.17, OS; HR: 0.48	Significantly higher rate of 2 year PFS with venetoclax plus rituximab than with a standard chemoimmunotherapy, with benefit observed in all subgroups analysed
Moreno et al. 2019 [48]	Americas, Europe, Asia and Australia	To compare the efficacy of the combination of ibrutinib plus obinutuzumab with chlorambucil plus obinutuzumab in first-line CLL/SLL	Rai stage III/IV	No	PFS; HR: 0.23	The progression-free survival benefit in the ibrutinib plus obinutuzumab group was particularly notable in patients considered to be in the high-risk group, which consisted of patients with del17p or TP53 mutation, del11q, or unmutated IGHV
Fischer et al. 2019 [49]	Europe, Americas and Oceania	To investigated fixed-duration treatment with venetoclax and obinutuzumab in patients with previously untreated CLL and coexisting conditions	Binet stage A, B and C	No	PFS; HR: 0.35, OS, HR: 1.24	targeted treatment with venetoclax–obinutuzumab was effective in previously untreated patients with CLL and coexisting conditions and resulted in a significantly higher percentage of patients with PFS than standard treatment with chlorambucil–obinutuzumab.
Shanafelt et al. 2019 [39]		To evaluate the efficacy and safety of treatment with ibrutinib in combination with rituximab, as compared with FCR, in previously untreated patients with CLL	Rai stage 0-II and III/IV	No	PFS; HR: 0.35 OS; HR: 0.17	Targeted therapy with ibrutinib improved 3-year PFS and OS as compared to standard chemoimmunotherapy in patients with previously untreated CLL
Sharman et al. 2020 [50]		To compare the efficacy of acalabrutinib with or without obinutuzumab against chlorambucil with obinutuzumab in patients with treatment naive CLL	Rai stage 0/I/II and III/IV	No	**Primary comparison** – PFS; HR: 0.1, OS; HR: 0.47 **Secondary comparison** PFS; HR: 0.2, OS: HR: 0.60	In patients with treatment-naive CLL, acalabrutinib with or without obinutuzumab improved progression-free survival over chemoimmunotherapy
Ghia et al. 2020 [41]	Americas, Europe, Middle East, Pacific Asia	To compare the efficacy and safety of acalabrutinib monotherapy versus investigator’s choice (I-R or B-R) in patients with R/R CLL	Rai stage III/IV	No	PFS; HR:0.31, OS; HR: 0.84	Acalabrutinib monotherapy significantly improved PFS compared with I-R or B-R in patients with R/R CLL. The benefit was shown in all prespecified subgroup analyses, including patients with high-risk genomic features, such as del(17p) plus TP53 mutation, del(11q), unmutated IGHV, as well as in prespecified analyses by baseline demographic and clinical characteristics

*CLL* Chronic lymphocytic leukaemia, *HR* Hazard ratio, *OR* Odd ratio, *PFS* Progression-free survival, *OS* Overall survival, *Del-* Deletion, *FCR* Fludarabine, cyclophosphamide and rituximab, *CIT* Chemoimmunotherapy, *EFS* Event-free survival, *ORR* Overall response rate, *RCC* Rituximab, cladribine and cyclophosphamide, *IGHV* Immunoglobulin heavy chain variable region gene, *CD-* Cluster differentiation, *R/R* Relapsed/refractory, *I-R* Ibrutinib plus rituximab, *B-R* Bendamustine plus rituximab

The geographic distribution of the included studies consisted of Europe, Americas, Asia, Australia (Table 1). The included studies comprised of 64% (*n* = 4 700) patients who were treatment-naïve, 11% (*n* = 815) of patients who were previously treated and 22.3% (*n* = 1 642) who were relapsed/refractory. In addition, 47% (*n* = 8) of the included studies reported on the Rai staging whereas 41% (*n* = 7) reported on Binet staging system. One study (6%) reported both Rai and Binet staging systems and another study (6%) did not specify the staging system used.

#### Prognostic factors in patients with CLL

In the included studies, prognostic factors were analysed before the start of treatment (Table 2). Overall, the studies comprised of 25.5% (*n* = 1 823) of patients who were 70 years or older, 55.7% (*n* = 3 984) of patients with an unmutated *IGHV* status, 17.4% (*n* = 1 245) with del11q, 6.8% (*n* = 489) with a del17p, 26.8% of the patients (*n* = 1 915) had del13q, and 3.9% (*n* = 264) had TP53 mutation. Notably, 6% (*n* = 429) patients were reported to have Trisomy 12. In the reported cell-based prognostic factors the included studies reported on ZAP-70 expression in 12.2% (*n* = 872) of the patients, and CD38 expression was reported in 12% (*n* = 863) of the included patients, 21.3 (*n* = 1 526) patients had elevated B2M levels (≥ 3.5 mg/L). In all, 36.7% (*n* = 2 625) of the included patients with CLL were in the advanced stage of the disease.

**Table 2:** Treatment arms and confirmed prognostic factors in studies included in the meta-analysis (*n* = 17)

Author, year	Study arms	Confirmed prognostic factors
Robak 2010 [16]	FCR vs FC	None
Hallek 2010 [17]	FCR vs FC	Del(17p), B2M, WCC, unmutated *IGHV*
Goede 2014 [15]	R-Chl vs Chl O-Chl vs Chl	None
Chanan-khan 2016 [46]	IBR vs Placebo-BR	None
Van Oers 2015 [51]	M-OFA vs OBS	None
Hillmen 2015 [47]	O-Chl vs Chl	None
Robak 2017 [42]	OFA + FC vs FC	None
Greil 2016 [44]	MR vs OBS	None
Dartigeas 2017 [45]	MR vs OBS	Unmutated IGHV
Robak 2018 [43]	MR vs OBS	Del(17p), Del(11q), elevated B2M
Woyach 2018 [14]	IR vs Ibr	Age, Del(17p), LDH
Seymour 2018 [40]	VenR vs BR	None
Moreno 2019 [48]	I-O vs O-Chl	None
Fischer 2019 [49]	Ven-O vs O-Chl	None
Shanafelt 2019 [39]	IR vs FCR	None
Sharman 2020 [50]	Acala vs Chl-O	None
Ghia 2020 [41]	Acala vs BR	None

*FCR* Fludarabine, cyclophosphamide plus rituximab, *Chl* Chlorambucil, *O-Chl* Obinutuzumab plus chlorambucil, *IBR* Ibrutinib plus bendamustine and rituximab, *M-OFA* Ofatumumab maintenance, *acala* acalabrutinib, *MR* rituximab maintenance, *OBS* Observation, *IR* Ibrutinib plus rituximab, *Ibr* Ibrutinib, *Ven-O* Venetoclax plus Obinutuzumab, *WCC* White cell count, *LDH* Lactate dehydrogenate, *IGHV* Immunoglobulin heavy chain variable region gene, *B2M* Beta-2-microglobulin, *Del-* Deletion

### Risk of bias and quality assessment

**Fig. 2:**
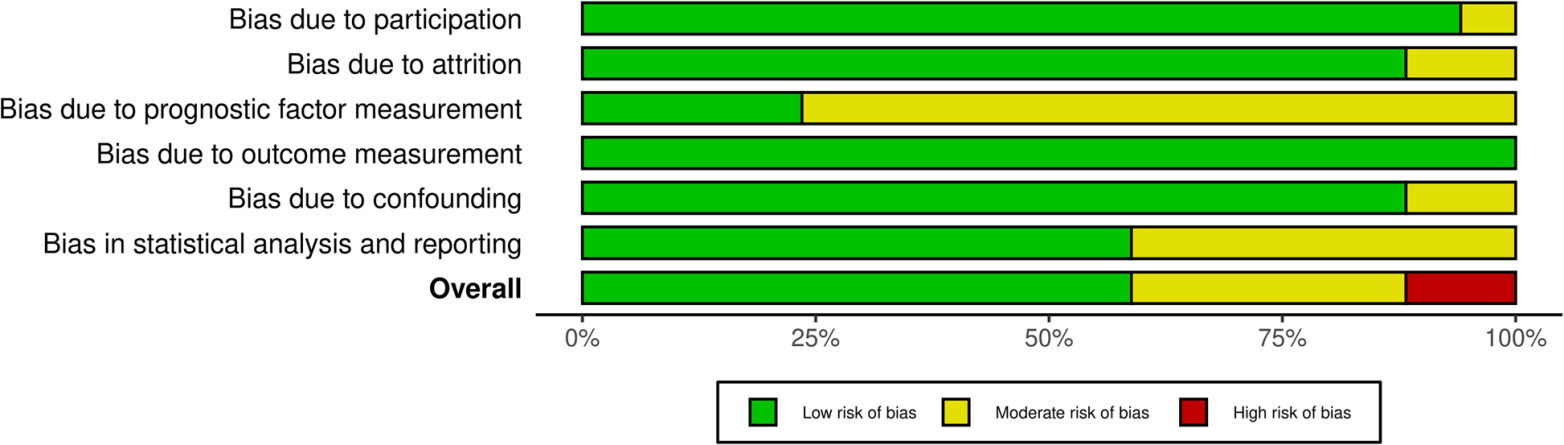
Risk of bias assessment of the prognostic factor studies

We assessed the quality of all included studies using the QUIPS tool for assessing risk of bias in prognostic factor studies [31]. The study-level risk of bias assessment is presented in Supplementary Table 2. Briefly, two studies were scored as high-risk [16, 41], five as moderate risk [39, 40, 47, 48, 50], whilst the rest were deemed to be at low risk of bias [14, 15, 17, 42–46, 49, 51]. Overall, the included studies were scored as low risk for study participation (k = 0.76, minimal agreement), and outcome measurement (k = 0.88, strong agreement), moderate risk for study attrition (k = 0.88, moderate agreement) and confounding measurement (k = 0.65, minimal agreement) and high risk for prognostic factor measurement (k = 0.90, strong agreement) and statistical analysis and reporting (k = 0.76, minimal agreement) (Fig. 2).

### Primary outcomes

#### Survival outcomes of patients with CLL receiving CIT containing anti-CD20

**Fig. 3:**
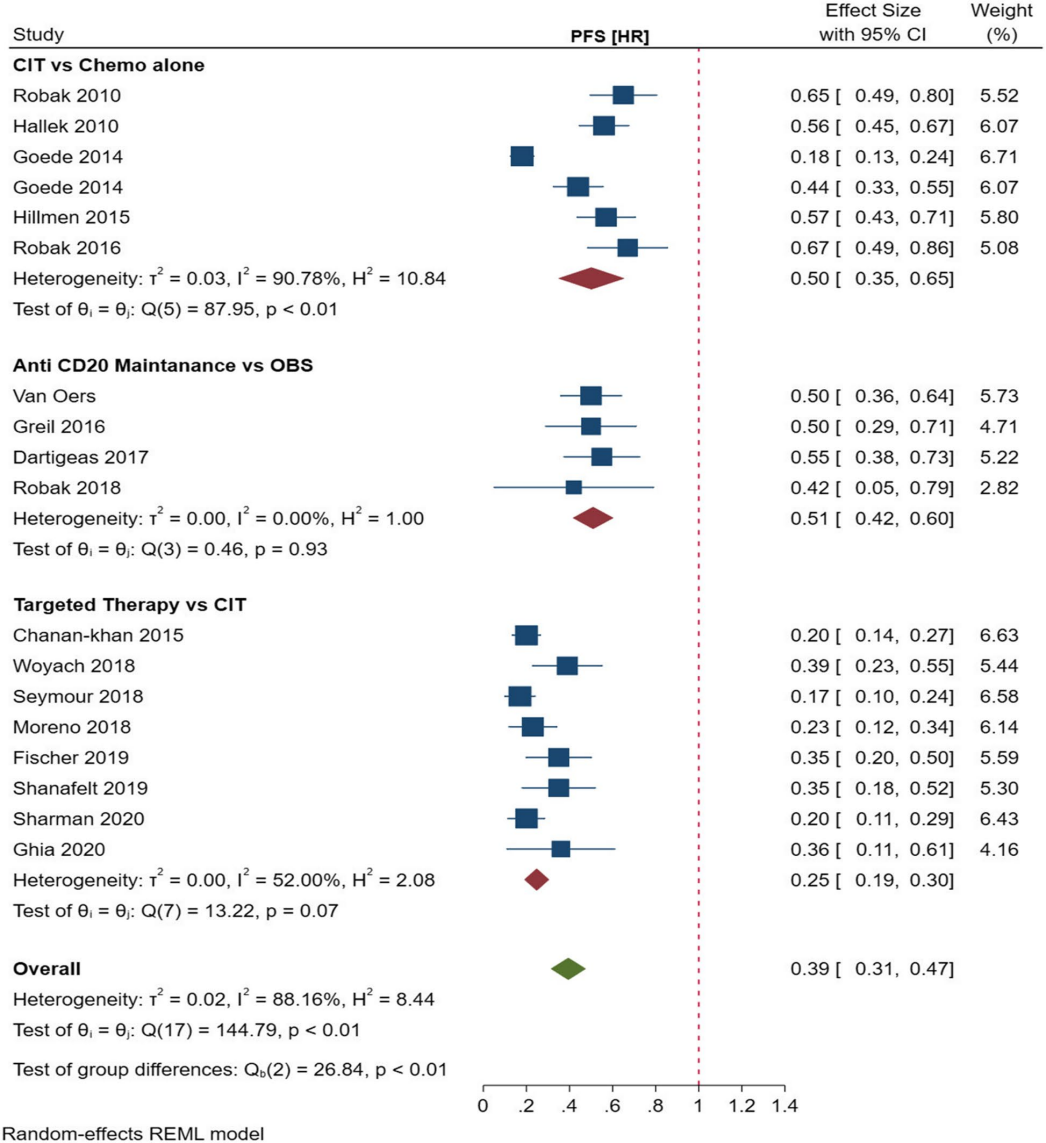
Meta-analysis of the hazards ratios (HR) for progression-free survival (PFS) for CLL patients treated with Anti-CD20 mAb containing CIT and standard chemotherapy alone or targeted therapy

A total of 5 studies [15–17, 42, 47] reported on an improved PFS in patients with CLL, when an anti-CD20 mAbs were concurrently used with standard chemotherapy. CIT in combination with anti-CD20 monoclonal antibodies, was associated with improved PFS (HR = 0.50 Cl [0.35–0.65], *p* < 0.01). There were high levels of heterogeneity (*I*^*2*^ = 90.78%) in the included studies. Overall, the pooled effect estimate showed no statistically significant difference in OS in patients with CLL treated with CIT and chemotherapy alone (*p* = 0.22) (Fig. 3).

#### Survival outcomes of patients with CLL on maintenance therapy with anti-CD20

A total of 4 studies [43–45, 51] reported on an improved PFS following maintenance therapy with anti-CD20 therapy as compared to patients who did not receive any treatment (observation group). The pooled effect estimate showed improved albeit non-significant PFS (HR = 0.51 [0.42–0.60], *p* = 0.93). There were no differences in OS between patients receiving maintenance therapy compared to those who were not on treatment. There were no significant differences in the pooled effect estimates (*p* = 0.96) and there were low levels of statistical heterogeneity amongst included studies, *I*^*2*^ = 0%.

#### Survival outcomes of patients with CLL receiving targeted therapy

In the meta-analysis, a total of eight studies [14, 39, 40, 46, 48–50] reported an improved PFS with novel targeted agents as compared to chemoimmunotherapy. Target therapy containing BTK and BLC2 inhibitors was associated with significantly improved PFS as compared to CIT (HR = 0.25 Cl [0.19–0.30], *p* = 0.07). OS data was available for seven studies [39–41, 46, 48–50]. Overall, targeted therapy was associated with improved OS (HR = 0.56 [0.33–0.80], *p* = 0.05). There were substantial levels of heterogeneity in the included studies (*I*^*2*^ = 51.67%).

**Fig. 4:**
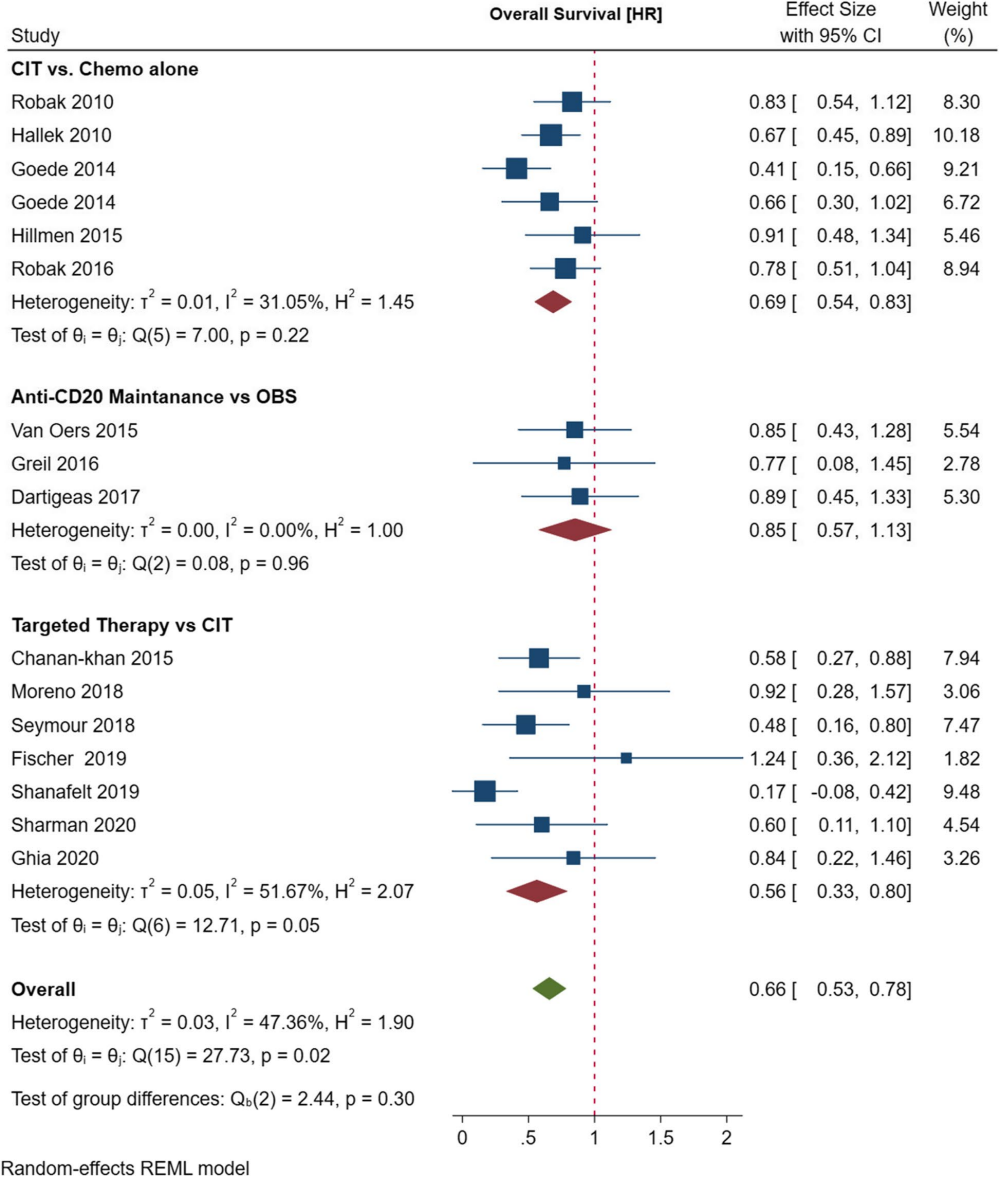
Meta-analysis of the hazards ratios (HR) for overall survival (OS) for CLL patients treated with Anti-CD20 mAb containing CIT and standard chemotherapy alone or targeted therapy

Overall, the meta-analysis shows that chemoimmunotherapy and maintenance therapy with anti-CD20 antibodies is superior to chemotherapy, and targeted therapy is superior to CIT in terms of PFS with HR = 0.39 [0.31–0.47], *p* < 0.01 and OS (HR = 0.66 [0.53–0.78], *p* < 0.02 (Fig. 4). There were high levels of heterogeneity on studies assessed for PFS (*I* = 88.16%).

#### Prognostic factors associated with poor patient outcomes in CLL patients

Prognostic markers ranged from host factors, such as age and cytogenetics, whereby 10 (58.8%) studies reported Del(17p) as a prognostic factor for PFS [14, 15, 17, 40–44, 47–50]. Two studies excluded patients with Del(17p) [45, 46] and in another study, del(17p) and del (11q) did not impact PFS [44]. Whereas 10 studies reported unmutated *IGHV* as a prognostic factor [17, 39–42, 45, 46, 48–50]. Trisomy 12 was identified as a prognostic factor in three studies [39, 42, 46] and TP aberrations was reported in four studies [40, 41, 48, 49].

The reported prognostic factors associated with early disease progression included elevated B2M levels (levels of ≥ 3.5 mg/L) [17, 43], thymidine kinase (concentration of 10 µ/L), white cell count (10 × 10^9^ per L) and ECOG PS of 2 [17] and advanced disease stage III/IV [17]. After adjusting for covariates, Del(17p), unmutated IGVH status and elevated B2M (Table 4).

## Discussion

We conducted a systematic review and meta-analysis of prognostic factors associated with poor survival in patients with chronic lymphocytic leukemia on CIT and novel targeted agents. The available data on the use of ICIs and targeted therapy in the management of CLL is limited to predominantly European and American populations (Table 1). The current study also highlights the lack of multi-ethnic RCTs with diverse population with CLL. The included studies reported on various candidate predictors of survival in patients with CLL on CIT and targeted therapy (Table 3).

**Table 3:** Characteristics of studies reporting on PFS/OS in patients with CLL on rituximab-containing regimens (*n* = 17)

Study	Source of data	Participant description	Sample size	Candidate predictors	Type of model	Model selection: stepwise selection, univariate *p*-values, no selection	Handling of continuous variables: retained as linear, categorised, dichotomised
Robak et al. 2010 [16]	RCT	Multicentre study conducted in Europe (88 centres, 17 countries). Patients ≥ 18 years with CD20 + CLL. Had received one prior line of treatment. ECOG PS of ≤ 1 and life expectancy of > 6 months	*N* = 552 (FCR: *n* = 276, FC: *n* = 276) Males: 67%	age, disease stage, creatinine clearance, and lymphocyte count	Cox regression (response rates) Logistic regression (prognostic factors)	Not stated	Not stated
Hallek et al. 2010 [17]	RCT	Multicentre study, enrolled treatment-naive patients with CLL (30—81 years). ECOG PS of 0–1, and a low comorbidity. Patients with absence of active disease were excluded	*N* = 817 (FCR: *n* = 408, FC: *n* = 409) Males: 74%	sex, age, disease stage, physical fitness, creatinine clearance, B2M, thymidine kinase, genomic aberrations, and *IGHV* mutational status	Cox proportional hazard model	Stepwise backward selection	Categorised
Goede et al. 2014 [15]	RCT	Multicentre study, enrolled previously untreated patients with CD20 + CLL, requiring treatment with coexisting conditions. Age ≥ 18 years	*N* = 589 (R-Clb: *n* = 233, O-Chl; *n* = 238, Clb: *n* = 118) Males: 62%	Genomic aberrations, *IGHV* mutational status	Not stated	Not stated	Categorised
Chanan-khan et al. 2016 [46]	RCT	Placebo-controlled, Multicentre study, enrolled patients ≥ 18 years with CLL requiring treatment. Had R/R disease following ≥ 1 previous lines of systemic therapy. ECOG status of 0–1. Patients with del17p were excluded	*N* = 578 (IBR: *n* = 289, Placebo-BR: *n* = 289) Males: 66%	ECOG performance status, ZAP70 expression, IGHV status, previous therapies, age, sex	Not stated	Not stated	Not stated
Hillmen et al. 2015 [47]	RCT	Multicentre study, enrolled untreated patients of any age, diagnosed with CLL with active disease requiring treatment. ECOG PS score of 0–2	*N* = 447 (O-Chl: *n* = 221, Chl: *n* = 226) Males: 63%	ECOG PS score, age, disease stage	Cox proportional hazards modelling	Not stated	Not stated
Robak 2017 [42]	RCT	Multicentre study, enrolled patients with relapsed but not refractory active CLL, ECOG PS score of 0–2 and a life expectancy of ≥ 6 months	*N* = 365 (OFA + FC: *n* = 183; FC: *n* = 182 Males: 60%	ECOG PS score, IGHV mutation status, B2m, del17p, del11q, Del13q, ZAP70, age, gender, disease stage	Not stated	Not stated	Not stated
Van Oers 2015 [51]	RCT	Multicentre study, included patients who were aged 18 years or older with a diagnosis of CLL in second or third complete or partial remission. WHO performance status of 0–2	*N* = 474 (OFA: *n* = 238; OBS: *n* = 236) Males = 67%	Del17p, Del13q, Del11q, unmutated IGHV, B2M	Not stated	Not stated	Not stated
Greil et al. 2016 [44]	RCT	Multicentre trial, enrolled patients ≥ 18 years with complete or partial response following previous first-/second-line rituximab-containing CIT. ECOG PS 0–2. Life expectancy of > 6 months	*N* = 263 (MR: *n* = 134, OBS: *n* = 129) Males: 71%	Sex, cytogenetic risk group, *IGHV* mutation status, and CD38 expression	Cox regression model	Univariate *p* values	Categorized
Dartigeas et al. 2017 [45]	RCT	Multicentre trial, enrolled fit, treatment naïve CLL patients aged ≥ 65 years requiring treatment	*N* = 409 (MR: *n* = 202, OBS: *n* = 207) Males: 66%	Age, sex, del(11q), Binet stage, *IGHV* mutational status, response to FCR	Cox regression model	Not stated	Not stated
Robak et al. 2018 [43]	RCT	Multicentre trial, enrolled patients ≥ 18 years old with previously untreated, progressive CLL	*N* = 66 (MR: *n* = 33, OBS: *n* = 33) Males: 68%	Age, sex, Rai stage, B2M level, chromosomal abnormalities and ZAP-70 or CD38 expression	Multivariate Cox’s proportional hazards regression model	Not stated	Categorized
Woyach et al. 2018 [14]	RCT	Multicentre trial conducted between Dec 2013—May 2016. Patients ≥ 65 years, untreated CLL	*N* = 365 (Ibr: *n* = 182, BR: *n* = 183) Males: 67%	ZAP-70 methylation status, Rai stage, del(17p13.1) or del(11q22.3)	Not stated	Not stated	Not stated
Seymour et al. 2018 [40]	RCT	Multicentre trial, enrolled patients 18 years or older with R/R CLL requiring treatment. Patients had received 1–3 previous treatments. ECOG PS of 0 or 1	*N* = 389 (VenR: *n* = 194; BR; *n* = 195) Males: 73.8%	Del17p, ECOG status, prior therapies, IGHV mutation status	Not stated	Not stated	Not stated
Moreno et al. 2019 [48]	RCT	Multicentre trial, included patients with untreated CLL aged 65 years or older, or younger patients with coexisting conditions. 96% of patients were White	*N* = 229 (I-O: *n* = 113; O-chl: *n* = 116) Males: 64.5%	Del17p, TP53 mutation ECOG status, IGHV mutation status	Not stated	Not stated	Not stated
Fischer et al. 2019 [49]	RCT	Multicentre trial, included patients who had previously untreated CD20 + CLL requiring therapy	*N* = 432 (Ven-O: *n* = 216; O-Chl: *n* = 216 Males: 66.9%	IGHV mutation status, TP53 deletion or mutation, coexisting conditions, disease stage	Cox proportional hazards analysis	Not stated	Not stated
Shanafelt et al. 2019 [39]	RCT	Multicentre trial, enrolled previously untreated patients with CLL/SLL who were 70 years of age or younger with no deletion of 17p13	*N* = 529 (IR: *n* = 354, FCR: *n* = 175) Males: 67.3%	ECOG performance status, disease stage, del11q, IGHV mutation status	Stratified Cox proportional-hazards models	Not stated	Not stated
Sharman et al. 2020 [50]	RCT	Multicentre study, including patients with treatment-naive CLL requiring treatment. Patients were ≥ 65 or ≥ 18 and < 65 with comorbidities	*N* = 535 (A-O: *n* = 179, Acala only: *n* = 179, O-C: *n* = 177) Males: 61%	Del17p, Del11q, unmutated IGHV, complex karyotype, TP53 mutation	Cox proportional model	Not stated	Not stated
Ghia et al. 2020 [41]	RCT	Multicentre study; included patients with relapsed/refractory CD20-positive CLL. ≥ 18 years with ECOG performance status ≤ 2. Must have received ≥ 1 prior systemic therapies for CLL. Patients who had previously received treatment with novel targeted agents were excluded	*N* = 310 (IR: *n* = 119, BR: *n* = 36, Acala: *n* = 155) Males: 67%	Del(17p), ECOG performance status score, lines of prior therapy, Del(11p)	Stratified cox regression	Not stated	Not stated

*Abbreviations: RCT* Randomised controlled trials, *WCC* White-cell count, *LDH* Lactate dehydrogenate, *CLL* Chronic lymphocytic leukaemia, *CD-* Cluster of differentiation, *PFS* Progression-free survival, *HR* Hazard ratio, *CIT* Chemoimmunotherapy, *R* Rituximab, *FCR* Fludarabine, cyclophosphamide and rituximab, *R-Clb* Rituximab plus chlorambucil, *G-Clb* Obinutuzumab plus Chlorambucil, *MR* rituximab maintenance, *OBS* Observation arm, *Ibr* Ibrutinib, *Ven* Venetoclax, *B2M* Beta-2-microglobulin, *Del-* Deletion, *IGHV* Immunoglobulin heavy variable region gene, *Del-* Deletion, *BTK* Bruton tyrosine kinase, *ECOG PS* Eastern cooperative oncology group performance status

Amongst the reported prognostic factors only one protein factor (β_2_-microglobulin) retained predictive value in patients with CLL on anti-CD20-containing CIT, after multivariable analysis. Only two other prognostic factors met our criteria for confirmed prognostic factors and these included, cytogenetic factors (deletion 17p, IGHV status). Notably, in our meta-analysis we pooled studies that reported on adjusted estimates and the levels of statistical heterogeneity were high (I^2^ > 70%) for the confirmed cytogenetic factors and for β_2_-microglobulin (Table 4). Interestingly, the value of β_2_-microglobulin as an independent prognostic marker has not been extensively assessed in patients with CLL on CIT and targeted therapy, although in a previous study its predictive value for treatment-free survival was retained after adjusting for factors such as CD38 expression and IGHV mutation status [52].

**Table 4:** Overview of confirmed prognostic factor included in the meta-analysis

Prognostic factors	Studies	Pooled HR	Lower limit	Upper limit	I^2^(%)	References
**Cytogenetic**						
Deletion 17p	3	3.39	-0.21	6.99	90.84	[14, 17, 43]
IGHV status	2	0.96	-0.07	1.99	94.02	[17, 53]
**Protein factors**						
β_2_ microglobulin	2	1.41	1.05	1.77	0	[17, 43]

*IGHV* Immunoglobulin heavy variable gene, *B2M* β_2-_microglobulin

The cut-off levels of B2M associated with poor prognosis remain unclear and in untreated CLL patients a value of 2 mg/L [54] while in our analysis B2M levels ≥ 3.5 mg/L [17, 43] were associated with disease progression in treated patients with CLL. Notably in the current analysis, we report on the retained predictive value of B2M in CLL patients on rituximab-containing CIT and maintenance therapy with rituximab. Future studies comprised of diverse patient populations are needed especially in minority ethnic groups to allow for validation of this prognostic marker in the era of CIT and novel targeted therapy. In the era of CIT, and chemotherapy-free CLL management, future studies evaluating the correlations between B2M levels and expression of CD20 and other immune checkpoints in patients with CLL, may assist in the stratification of patients who are most responsive to immunotherapy.

To the best of our knowledge this systematic review and meta-analysis provides the first analysis of prognostic factors in anti-CD20-containing CIT and targeted therapy. The current study has several limitations, firstly these findings are mainly derived from American and European populations. This limits the extrapolation of these findings into other low-to-middle income countries. Lastly, due to the low number of studies reporting on these prognostic factors in patients with CLL on CIT and targeted therapy, we could not explore the sources of heterogeneity in a subgroup analysis based on the potential differences in disease stage and duration of follow-up.

## Conclusion

A plethora of novel prognostic factors have been described in untreated patients with CLL. However, in the era of CIT there is a lack of adequate studies exploring the predictive value of the conventional and novel prognostic factors in a multi-ethnic cohort of patients with CLL. In this systematic review and meta-analysis of prognostic factors, classical cytogenetic factors such as deletion 17p retained predictive value in patients with CLL on CIT. Lastly, the white cell count and conventional prognostic markers such as B2M and LDH levels were also regarded as confirmed prognostic factors in patients with CLL on rituximab containing CIT. These factors should be included in future prognostic factors in the era of CIT and chemotherapy-free era of CLL patient management.

## Supplementary Information

**Additional file 1: Supplementary Table 1. **Search strategy.

**Additional file 2: Supplementary Table 2. **Risk of bias assessment of individual studies using the QUIPS tool.

## Data Availability

All data generated or analysed during this study are included in this publication.
