# Supplementary material for: Prognostic markers in patients with chronic lymphocytic leukaemia on targeted therapy, chemoimmunotherapy with anti-CD20 monoclonal antibody: a systematic review and meta-analysis of prognostic factors
Source: BMC Cancer. 2022 Nov 25;22:1218. doi: 10.1186/s12885-022-10223-0 (PMC9701011; doi:10.1186/s12885-022-10223-0)
Supplement: Supplementary file 1 — Additional file 1: Supplementary Table 1. Search strategy. [file 12885_2022_10223_MOESM1_ESM.pdf]

Supplementary Table 1: Search strategy

Database	Search terms	Synonyms/associated terms	Hits
Medline	Concept 1<br>"Chronic lymphocytic leukemia"[MeSH]	CLL	23 862
	Concept 2<br>“Prognosis” [MeSH]	Prognostication, prognostic, prediction	794,891
	Concept 3<br>“Rituximab” [MeSH]	Rituxan, anti-CD20 mAb	5,346
	Concept 4<br>“Obinutuzumab”	anti-CD20 mAb	411
	Concept 5<br>Ofatumumab	anti-CD20 mAb	368
	Concept 6<br>Ibrutinib	BTK-inhibitor	1 687
	Concept 7<br>Venetoclax	BCL-2 inhibitor	1 107
	Concept 8<br>Acalabrutinib	BTK inhibitor	147
	Concept 9<br>Idelalisib	PI3K inhibitor	359

Combined concepts

Concept 10= Combine 1 & 2	Hits
("Leukemia, Lymphocytic, Chronic, B-Cell"[Mesh]) AND "Prognosis"[Mesh]	**4 462**
**Combined concept 10 & 3**	
("Leukemia, Lymphocytic, Chronic, B-Cell"[Mesh]) AND "Prognosis"[Mesh]) AND "Rituximab"[Mesh]	**476**
**Combined concept 10 &4**	
"Leukemia, Lymphocytic, Chronic, B-Cell"[Mesh]) AND "Prognosis"[Mesh]) AND "obinutuzumab" [Supplementary Concept]	**47**
**Combined concept 10 & 5**	
("Leukemia, Lymphocytic, Chronic, B-Cell"[Mesh]) AND "Prognosis"[Mesh]) AND "ofatumumab" [Supplementary Concept]	**74**
**Combined Concept 10 & 6**	
("Leukemia, Lymphocytic, Chronic, B-Cell"[Mesh]) AND "Prognosis"[Mesh]) AND "ibrutinib" [Supplementary Concept]	**269**
**Combined Concept 10& 7**	
("Leukemia, Lymphocytic, Chronic, B-Cell"[Mesh]) AND "Prognosis"[Mesh]) AND Venetoclax	**146**
**Combined Concept 10 & 8**	
("Leukemia, Lymphocytic, Chronic, B-Cell"[Mesh]) AND "Prognosis"[Mesh]) AND Acalabrutinib	**31**
**Combined Concept 10 & 9**	
"Leukemia, Lymphocytic, Chronic, B-Cell"[Mesh]) AND "Prognosis"[Mesh]) AND Idelalisib	**132**
