# Supplementary material for: Prognostic markers in patients with chronic lymphocytic leukaemia on targeted therapy, chemoimmunotherapy with anti-CD20 monoclonal antibody: a systematic review and meta-analysis of prognostic factors
Source: BMC Cancer. 2022 Nov 25;22:1218. doi: 10.1186/s12885-022-10223-0 (PMC9701011; doi:10.1186/s12885-022-10223-0)
Supplement: Supplementary file 2 — Additional file 2: Supplementary Table 2. Risk of bias assessment of individual studies using the QUIPS tool. [file 12885_2022_10223_MOESM2_ESM.pdf]

Supplementary Table 2. Risk of bias assessment of individual studies using the QUIPS tool.

References	Study participation	Study attrition	Prognostic factor measurement	Outcome measuremen t	Study confounding	Statistical analysis & reporting	Overall
Robak 2010	Low	Low	Moderate	Low	Moderate	Moderate	High
Hallek 2010	Low	Low	Low	Low	Low	Low	Low
Goede 2014	Low	Low	Moderate	Low	Low	Low	Low
Hillmen 2015	Low	Low	Moderate	Low	Low	Moderate	Moderate
Chanan-khan 2015	Low	Low	Moderate	Low	Low	Low	Low
Robak 2016	Low	Low	Moderate	Low	Low	Low	Low
Greil 2016	Low	Low	Moderate	Low	Low	Low	Low
Dartigeas 2017	Low	Low	Moderate	Low	Low	Low	Low
Robak 2018	Moderate	Low	Low	Low	Low	Low	Low
Woyach 2018	Low	Low	Low	Low	Moderate	Low	Low
Seymour 2018	Low	Low	Moderate	Low	Low	Moderate	Moderate
Moreno 2018	Low	Low	Moderate	Low	Low	Moderate	Moderate
Fischer 2019	Low	Low	Moderate	Low	Low	Low	Low
Shanafelt 2019	Low	Low	Moderate	Low	Low	Moderate	Moderate
Sharman 2020	Low	Low	Moderate	Low	Low	Moderate	Moderate
Ghia 2020	Low	Moderate	Moderate	Low	Moderate	Moderate	High

Low = All domains were classified as having Low RoB, or up to one moderate RoB.

Moderate = mainly Low RoB-domains and up to two moderate RoB.

High = ≥ one domain with high RoB or ≥ three moderate RoB
